# Reproductive Parameters of the Dogo Argentino Bitch

**DOI:** 10.1155/2013/495975

**Published:** 2013-10-28

**Authors:** Marina Caffaratti, Griselda González, Nora Gorla, Corina Guendulain

**Affiliations:** ^1^Departamento de Clínica Animal, Facultad de Agronomía y Veterinaria, Universidad Nacional de Río Cuarto, Ruta Nac. 36 Km 601, Córdoba, CP 5804BYA Río Cuarto, Argentina; ^2^Facultad de Agronomía y Veterinaria, Universidad Nacional de Río Cuarto-CONICET, Argentina

## Abstract

The Dogo Argentino (DA) is the first and only breed from Argentina recognized worldwide. Although its morphologic features have been well established, its normal reproductive parameters are not clearly known. The aim of this study was to determine the main DA bitch reproductive parameters. One hundred and forty-nine surveys were obtained from breeders from Córdoba province, Argentina: one for each intact DA bitch from 1 to 14 years old. The DA bitch reached puberty at an average of 8.93 months. The mean duration of vulval bleeding found in this study was 11.11 days. The clinical signs characteristic for proestrous-estrous were vulval edema (89.93%), bleeding during the time of mating (32.21%), holding the tail to the side (95.30%), and docility during mating (85.91%). DA bitches had a whelping rate of 84%. Out of 299 pregnancies, 89.30% exhibited a normal parturition, 6.69% presented dystocia, 2.68% needed Cesarean section, and 1.34% aborted. In conclusion, the reproductive parameters of the DA bitch are similar to those identified for other large breeds. DA often showed a prolonged vulval bleeding longer than proestrus. Its high whelping rate, its low incidence of dystocia, and its good maternal ability define the DA as a good reproductive breed with normal reproductive functions.

## 1. Introduction

The Dogo Argentino (DA) is so far the only breed from Argentina recognized by the Fédération Cynologique Internationale (1973). Even though it is known as a big-game hunting dog, it is now also used as companion and guard dog. Its morphologic features have been very well established; however, its normal reproductive parameters are not clearly known. This knowledge is essential to achieve a proper reproductive management, to determine the ideal time of mating or artificial insemination, to avoid unwanted pregnancies, to estimate whelping date, and to diagnose many pathologies. 

Domestic dogs are monoestrous and typically nonseasonal breeders, with large individual and breed-specific variations [1]. They reach puberty variably at 6 to 14 months of age, with means positively correlated with breed size [2]. The average of the interestrous interval is 6 months, it ranges from 4 to 12 months depending on breed, age, and individual [3]. The proestrous duration is highly variable, from 6 to 11 days, with an average of 9 days [4]. This stage is characterized by male attraction, nonreceptive bitches, and vulval bleeding. Pseudopregnancy is a physiological syndrome which may occur during diestrous. Its signs are very similar to those typical of pregnant bitches, such as mammary development, appetite and weight increase, abdominal enlargement, lactation, signs of impending birth, nesting behaviour, mothering of objects, and, sometimes, abdominal contractions. This behaviour is more frequent in animals kept in close contact with their owners [5], though its exact prevalence in any breed remains unknown.

Bitch pregnancy rate by natural mating depends on many factors and has been reported to be around 78% [6] or even as high as 97% [7]. Gestation duration can be estimated from 55 to 72 days from the first mating [4]. This parameter is useful to estimate the parturition date, to schedule an elective Cesarean section, to manage the pregnancy, and to diagnose dystocia. The latter is one of the most important pathologies of parturition and occurs in approximately 5% of bitches [8]. When this situation cannot be resolved by medical procedure, Cesarean section is indicated. The Cesarean-section incidence has not been established yet and depends strongly on the breed [8, 9].

There are some studies of litter size based on data from different kennel clubs and breeders [8, 9]; however, it has not been established in DA. There are many suggested factors that affect litter size, but breed size appears to be the strongest factor [10].

A proper maternal behaviour is crucial for neonatal survival and includes breastfeeding, recovery of the puppies, grooming, and protection [11]. Although this is an instinctive behaviour, it differs among individuals and may depend on breed.

There is little information about the DA, and, to the best of our knowledge, there are no studies related to its reproduction. Thus, the aim of this study was to determine the main DA bitch reproductive parameters.

## 2. Material and Methods

One hundred and forty-nine surveys (80% of 187 surveys made) were obtained from records of 65 breeders from Córdoba province, Argentina, during 2010-2011: one for each intact bitch from 1 to 14 years of age (4.8 years on average; ±2.6 SD). These bitches were registered as pure breed. The surveys were made personally and consisted of 17 questions about different physiological variables: age of the first heat (97.3% responded), time between heats (97.3% responded), proestrous-estrous signs (100% responded), frequency of pseudopregnancy (100% responded) and its signs (100% responded out of those positive to pseudopregnancy), number of pregnancies (100% responded; 85.9% pregnant at least once), number of matings (89.3% responded), whelping type (100% responded), gestation duration (89% responded), litter size (95.2% responded), male/female rate in the litter (75.2% responded), and maternal ability (99.2% responded). The latest was evaluated by asking the owner to give a score from 1 to 3, where grade 3 (good) corresponded to those bitches with normal maternal behaviour: long lying on the puppies, breastfeeding, intensive grooming, recovery, and protection. Grade 1 (bad) corresponded to those bitches which rejected puppies, stayed for short terms in the nest, pressed the puppies, and did not allow breastfeeding. Grade 2 (regular) represented an intermediate behaviour. A retrospective data collection was carried out, and InfoStat 2011 [12] software was used to make a descriptive data analysis.

## 3. Results and Discussion

This investigation is based on information provided by DA breeders and owners through personal surveys. In spite of the fact that most of them kept a record of their breeding activities, not all the survey questions could be answered. 

**Figure 1:**
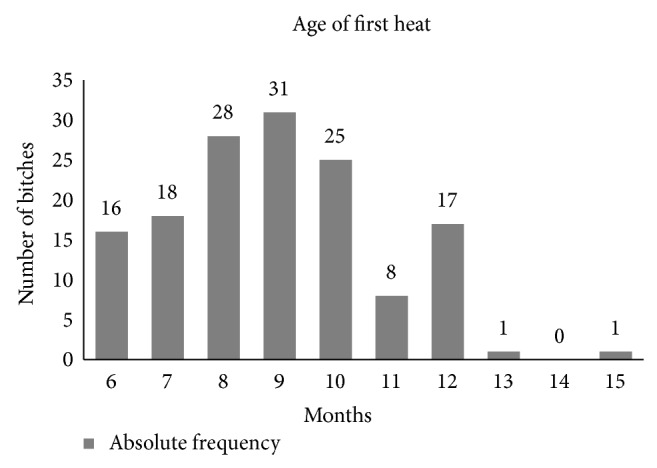
Absolute frequency of the age of first heat in DA bitches. Most of the bitches reached puberty at 9 months.

In bitches, puberty can be recognized by the appearance of the first estrous cycle. Generally, this happens some months after they get their adult weight and height; however, it varies across breeds. For example, in Beagles it occurs between 7 and 10 months, whereas the DA bitch reached puberty (age at first detected estrous) at the age of 8.93 (±1.87 SD) months (Figure 1), which is in line with the findings of Concannon [2], who mentions a positive correlation between age at first estrous and the size of the breed.

The interestrous interval is very variable between breeds and also within individuals of the same breed. The 83.89% of DA evaluated bitches had their cycle regularly every 6.01 months on average (±0.5 SD), 13.42% showed irregular cycles (variable periods from 5 to 8 months), and in 2.69% of the bitches the owners could not provide this information. This interestrous interval is in agreement with previous findings for most breeds, except for German Shepherd dog which has short intervals from 4 to 4.5 [13] and Basenji with interestrous intervals as long as 12 months.

Vulval bleeding is a characteristic proestrous sign with a duration of 6 to 11 days (9 days on average) [4]. The mean vulval bleeding found in this study was 11.11 days (based on 138 bitches). The characteristics for proestrous-estrous signs analyzed were vulval edema (89.93%), bleeding during mating (32.21%), holding the tail to the side (95.30%), and docility during mating (85.91%). Although some bitches may have haemorrhagic discharge during all the proestrous, estrous, and diestrous [4], this sign is not common. Due to the high percentage of DA bitches (32.21%) which were receptive to mounting even when still bleeding, it is important to consider that this sign remains during estrous. Thus, it cannot be used as a reliable parameter to determine the optimal time of mating. Moreover, it could be necessary to make serial vaginal cytology in order to confirm if, besides prolonged vulval bleeding, DA bitches have longer proestrous than the normal average of 9 days.

Pseudopregnancy or pseudocyesis is a physiological condition in which the female shows similar signs to pregnancy. Although its exact prevalence is not known, it is estimated between 50 and 75% [14]. However, in DA bitches, the value recorded was 30.2%. The evaluated signs of this stage were nesting (64.44%), mammary enlargement (93.33%), milk secretion (84.44%), and weight gain (42.22%). Even though the occurrence of pseudopregnancy was not high, the frequent mammary enlargement and milk secretion in these bitches should be considered in order to prevent and treat the occasional happening of mastitis which is prone in these situations.

A hundred and thirty-three of the total bitches had at least one mating during their lifetime and showed a whelping rate of 84%. This value agrees with the normal range of 80 to 95% shown in canines [15]. Despite the fact that a few owners made tests (vaginal cytology and serum progesterone) to determine the ideal mating date, the whelping rate found was higher than the one described in a Drever kennel, where the whelping average recorded was 78.6% [9].

The gestation duration can be estimated from 55 to 72 days from the first mating [4]. This information is in agreement with the data obtained in the present investigation based on 295 full-term pregnancies, where the mean gestation duration from the first day of mating was 61.12 days (±1.68 SD) and the range was 57 to 68 days. This parameter changes according to the breed and litter size: the duration of pregnancy is 0.25 days longer for each puppy less than the average for the breed [9]. Also, litters of four or fewer tend to have longer gestation duration [16].

**Figure 2:**
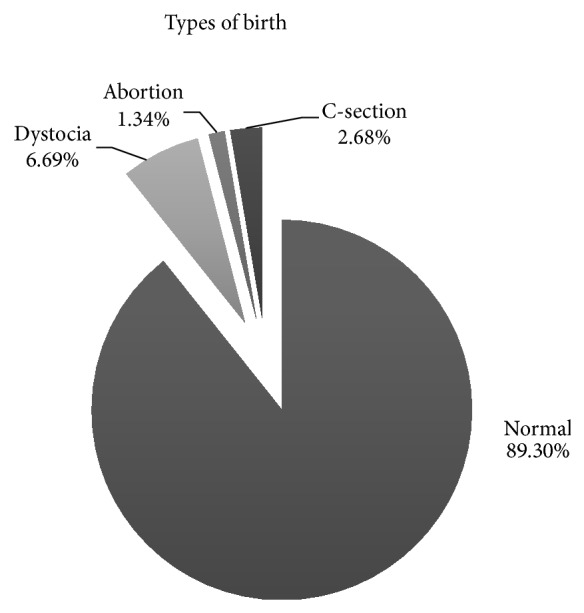
Types of birth. Percentage of each type of birth or abortion in DA bitches.

From 299 pregnancies (based on 128 bitches), 89.30% exhibited normal parturition, 6.69% presented dystocia, 2.68% needed Cesarean section due a dystocia, and 1.34% aborted (Figure 2). The incidence of dystocia found in DA was slightly higher than the normal average (5%) [17] but lower than in the Golden Retriever (9.1%), Pekingese (85.7%) [18], and Boxer (32%) breed [8]. The low frequency of dystocia in DA compared with these breeds is probably due to its size and body characteristics (mesomorphic and mesocephalic), which makes delivery easier than other breeds. This is proved by the low incidence of Cesarean section (2.68%) in relation to Drever (5.36%) [9] and Boxer (28.7%) breeds [8].

The bigger the breed is, the larger the average litter size will be. The mean litter size found in this study was 8.12 (±3.44 SD), which is in agreement with Feldman and Nelson [4], who found an average of 8 to 10 puppies, but differs from Borge et al. [10], who recorded a mean of 6.9 puppies in large breeds. As for the percentage of male puppies observed in this study (50.8%), it is similar to the average found in Drever breed (50.5%) [9] as well as that found in other studies which report a mean of 50.6% [19].

**Figure 3:**
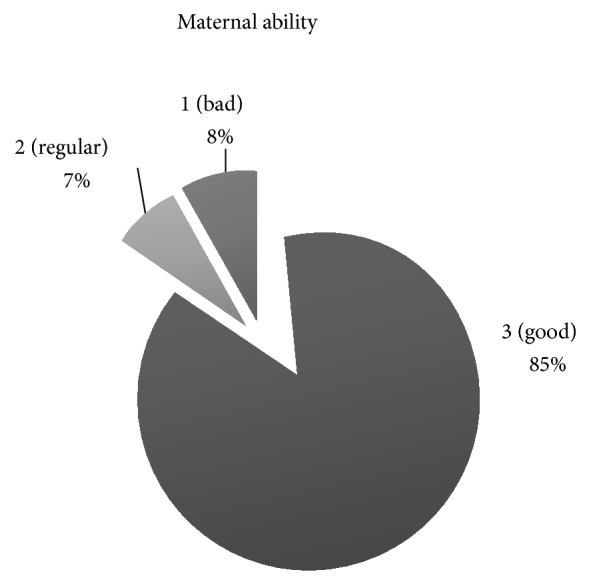
Maternal ability. Percentage of the scores given to the bitches by their owners: 3: good; 2: regular; and 1: bad maternal behavior.

Other data collected were related to maternal ability (Figure 3). Even though the DA bitch could be considered a good mother due to the high percentage of bitches classified by their owners in grade 3, this parameter has not been studied in other breeds to allow for comparisons.

## 4. Conclusions

The reproductive parameters of the DA bitch are in agreement with those identified for other large breeds. DA often showed a prolonged vulval bleeding longer than proestrus. Its high whelping rate, its low incidence of dystocia, and its good maternal ability define the DA as a good reproductive breed with normal reproductive functions.
